# “When I Don’t Have a Cigarette It’s Helpful, but It Really Don’t Satisfy:” Qualitative Study of Electronic Nicotine Delivery Systems (ENDS) Use among Low-Income Smokers

**DOI:** 10.3390/ijerph19031157

**Published:** 2022-01-20

**Authors:** Claire A. Spears, Dina M. Jones, Cherell Cottrell-Daniels, Hala Elahi, Courtney Strosnider, Jackie Luong, Scott R. Weaver, Terry F. Pechacek

**Affiliations:** 1 Department of Health Policy and Behavioral Sciences, School of Public Health, Georgia State University, Atlanta, GA 30303, USA; halaelahi@gmail.com (H.E.); cstrosnider@gsu.edu (C.S.); jackmluong@gmail.com (J.L.); tpechacek@gsu.edu (T.F.P.); 2 Center for the Study of Tobacco, Department of Health Behavior and Health Education, College of Public Health, University of Arkansas for Medical Sciences, Little Rock, AR 72205, USA; dmjones2@uams.edu; 3 Department of Health Outcomes and Behavior, Division of Population Sciences, Moffitt Cancer Center, Tampa, FL 33617, USA; Cherell.Cottrell-Daniels@moffitt.org; 4 Department of Population Health Sciences, School of Public Health, Georgia State University, Atlanta, GA 30303, USA; srweaver@gsu.edu

**Keywords:** cigarettes, e-cigarettes, electronic nicotine delivery systems, income, disparities, qualitative

## Abstract

Background: Little is known about the use of electronic nicotine delivery systems (ENDS) among low-income adult cigarette smokers, who experience severe tobacco-related health disparities. Methods: This study conducted interviews to examine experiences and perceptions associated with ENDS use among predominantly low-income adult smokers (*n* = 30; mean age 30.2 ± 12.9; 60% male, 46.7% African American, 30% white, 10% more than one race; 76.7% annual household income ≤USD 24,000). Interviews were transcribed verbatim and coded in NVivo 11. Results: Overall, participants reported complementing rather than substituting their smoking with ENDS use (e.g., using ENDS only when smoking is not allowed). Predominant reasons for vaping were convenience, smoking reduction/cessation, stress management, social acceptability, lower long-term costs than smoking, and appealing flavors. Common reasons for not switching to exclusive vaping were that ENDS did not satisfy cigarette cravings and concerns about ENDS health effects. Participants indicated higher likelihood of switching to exclusive ENDS use if the products were more affordable, perceived as substantially less harmful, tasted and felt more like smoking a cigarette, and more effective for reducing cravings. Conclusions: Continued research is needed to maximize any harm reduction potential of ENDS and ensure that these products do not contribute to worsening health disparities.

## 1. Introduction

Adults with low socioeconomic status (SES) exhibit disproportionately high rates of cigarette smoking, have greater difficulty quitting, and experience profound tobacco-related health disparities [1,2,3,4]. Although smoking prevalence has declined in the general U.S. population, smoking rates remain disproportionately high among low-SES adults [5,6]. There is an urgent need for effective strategies to reduce the harm of tobacco among low-SES individuals. The U.S. market for electronic nicotine delivery systems (ENDS; i.e., e-cigarettes) has grown over the past decade [7]. The current scientific consensus is that ENDS use (i.e., “vaping”), while not harmless, is likely less harmful than smoking combustible cigarettes [8]. ENDS could potentially reduce population harm from tobacco if current smokers switch completely to ENDS. A 2021 systematic review [9] concluded that there was “moderate-certainty evidence” that e-cigarettes containing nicotine are more effective for smoking cessation than e-cigarettes without nicotine and nicotine replacement therapy. However, comparisons between e-cigarettes and usual care/no treatment were unclear, and estimates were imprecise due to the small number of randomized controlled trials. In another systematic review, the efficacy of e-cigarettes for smoking cessation was questioned, particularly when commercially available e-cigarettes were used in self-initiated cessation attempts [10]. Moreover, prolonged dual use of ENDS and cigarettes is associated with health harms including respiratory symptoms [11,12,13]. Complete switching from cigarettes to ENDS is necessary to reap potential health benefits of harm reduction [8,14].

Relatively little is known about ENDS use and perceptions specifically among low-SES adults. Surveys on the prevalence of ENDS use by SES have been mixed, but limited data suggest that adults with low education may be less aware of and less likely to use ENDS [15]. Conversely, 2019 U.S. nationally representative data [16] indicate that adults with household income < USD 35,000 (vs. ≥USD 100,000) were more likely to use e-cigarettes every day or some days (5.0% vs. 3.8%). Similarly, adults without health insurance (7.2%) were more likely to use e-cigarettes than those with private insurance (4.3%) [16]. However, examinations specifically among cigarette smokers are needed. For example, based on 2016–2017 U.S. nationally representative surveys, among current cigarette smokers, those living below the poverty level were less likely to have ever used ENDS, and those with lower education were less likely to currently use ENDS [17].

Low-SES smokers who are otherwise unable to quit smoking arguably have the most to gain from potential harm reduction products such as ENDS, provided they can switch completely to ENDS. However, health disparities could be exacerbated if higher-SES smokers successfully use ENDS for harm reduction, but low-SES smokers are less likely to use ENDS to completely quit smoking cigarettes. Furthermore, low-SES smokers may be less likely to afford advanced ENDS devices or receive appropriate educational/healthcare services that help them use ENDS to effectively quit smoking [18]. Some data suggest that among cigarette smokers, those with lower education or income are less likely to switch to exclusive ENDS use [19,20]. A systematic review of e-cigarettes for smoking cessation among priority populations (including people who are homeless, have mental health conditions, or misuse substances) determined that there is not sufficient evidence to conclude whether e-cigarettes are effective for smoking cessation in these subgroups [21]. Since most studies have been relatively small and uncontrolled, more research is needed to determine the utility of ENDS for smoking cessation in low-SES populations, particularly when commercially available e-cigarettes are used in self-initiated cessation attempts [10]. Moreover, ENDS use patterns, perceptions, and motives are largely unknown among low-SES dual users of cigarettes and ENDS. Accordingly, this study utilized in-depth qualitative interviews to examine the experiences and perceptions associated with ENDS use among predominantly low-income adult smokers.

## 2. Materials and Methods

### 2.1. Participants

Participants were recruited through flyers in low-income areas (e.g., community health centers serving uninsured and under-insured individuals, train/bus stops near downtown Atlanta, Georgia) in September/October 2018. Inclusion criteria were age 18–65; ability to speak, read, and write in English; ownership of a smartphone with monthly plan (for ecological momentary assessments described elsewhere); be a current (every day/some days) cigarette smoker and current (every day/some days) ENDS user. Cigarette smoking status was assessed with the question: “Do you now smoke cigarettes every day, some days, rarely, or not at all?” To assess ENDS use, participants were first provided a brief description of electronic vapor products, with example types and brands. They were then asked, “Do you currently use electronic vapor products, also known as e-cigarettes, vapes or vaping, or electronic nicotine delivery systems?”. If yes, they were asked, “How often do you typically use these products?” with response options “every day”, “some days”, “rarely”, or “not at all”. Individuals who indicated that they currently smoked cigarettes every day or some days and also currently used ENDS every day or some days were included. Exclusion criteria were current pregnancy/lactation or another household member in the study.

### 2.2. Procedures

After telephone screening, participants attended an in-person session to provide written informed consent and complete questionnaires on demographics, tobacco use, and interest in quitting smoking [22]. Participants also rated the importance of 18 reasons for using ENDS based on the Population Assessment of Tobacco and Health study [23], using a 7-point scale (0 = not at all important, 6 = very important). Reasons included appealing flavors, ENDS harm perceptions, quitting smoking, use in smoke-free environments, smell, feels drawn to smoking a regular cigarette, and use of ENDS to relax/manage stress and emotions.

Participants engaged in in-depth individual interviews beginning with: “What thoughts, images, and feelings come to your mind when you think about electronic vapor products, also commonly referred to as e-cigarettes, electronic nicotine delivery systems, vapes, and many other terms?” Participants were asked their reasons for using ENDS and what they enjoyed and did not enjoy about ENDS, including perceived benefits and risks. They were also asked about situations in which they were more likely to vape vs. smoke and vice versa. Regarding ENDS and quitting smoking, participants were asked: “Do you think you smoke more cigarettes or less cigarettes since you started using electronic vaping products?” and “What factors would make you more likely to switch completely from cigarettes to electronic vapor products?” Interviews were conducted by trained student research assistants. The number of interviews was determined based on saturation [24].

Interviews were audio-recorded (without participants’ names), transcribed verbatim, and coded with NVivo 11 using both inductive and deductive approaches [25,26]. First, the lead author and a team of six coders reviewed the transcripts and developed an initial list of codes based on the interview guide and themes emerging from the transcripts. Next, the six coders independently coded the same transcript and met as a group with the lead author to review inconsistencies and refine the coding scheme. All coders then independently coded three additional transcripts and met as a group with the lead author to finalize the coding manual. All remaining transcripts were each independently coded by two coders. Throughout this process the group met regularly to resolve inconsistencies, with final decisions made by the first and second authors.

A total of 78 individuals completed the phone screen, 45 of these were eligible, and 31 attended the baseline in-person session to enroll and complete the interview. One participant was removed from analyses due to being an SES outlier (annual household income > USD 84,000). Participants were compensated USD 40 for completing the baseline questionnaire and interview session. The study was approved by the Georgia State University Institutional Review Board (protocol H18610).

## 3. Results

The analytic sample included 30 adults (mean age 30.2, SD = 12.9; 60.0% male, 46.7% African American, 30% white; see Table 1). SES is typically indicated by measures of income and education [4], and our sample is consistent with low SES in the U.S. based on both indicators (i.e., 76.7% annual household income ≤USD 24,000, with 100% annual household income below the U.S. median [27]; 96.6% with education less than bachelor’s degree). Most (*n* = 22; 73.3%) were daily cigarette smokers. Motivation to quit smoking was mixed, with 36.7% intending to quit smoking someday but not in the next year, 23.3% in the next year, and 20% in the next 6 months. Thirteen currently used ENDS daily and the majority (90.0%) used rechargeable ENDS. Most (≥70%) used tank systems and refillable ENDS. In the past 30 days, the majority (90.0%) used ENDS in situations where they could not smoke regular cigarettes and many used tobacco products other than cigarettes and ENDS (e.g., 76.7% currently used little cigars and cigarillos [LCCs]).

### 3.1. Reasons for ENDS Use

Participants gave the highest ratings for “I could use them in places where regular cigarette smoking isn’t allowed” (M = 5.14, SD = 1.25), followed by “Electronic vapor products could help me reduce the number of regular cigarettes I smoke” (M = 4.60, SD = 1.67), “When I’m feeling irritable, using electronic vapor products helps me relax” (M = 4.30, SD = 1.80), and “Electronic vapor products don’t smell” (M = 4.24, SD = 2.06). The reason with the lowest rating was “Using an electronic vapor product feels similar to smoking a regular cigarette” (M = 1.93, SD = 1.98). Qualitative findings were consistent with these ratings, and themes are shown in Table 2. Participants described that convenience; efforts to reduce or quit cigarette smoking; stress management/relaxation; and discreteness/social acceptability were primary reasons for ENDS use. Several also liked ENDS flavors and discussed the lower cost of ENDS compared to smoking.

#### 3.1.1. Convenience

Participants commonly used ENDS indoors and when cigarettes were not available or allowed:

“I can actually use it in my home. My significant other does not smoke, but she allows me to vape inside, so if I don’t feel like going outside, I can use that and just get a little nicotine…. If I’m in a restaurant or mall that doesn’t allow smoking, sometimes don’t feel like goin’ outside, you can hit the vape and get away with it”.

(45-year-old multi-race male)

#### 3.1.2. Smoking Reduction or Cessation

Participants described using ENDS in attempt to reduce or quit smoking:

“I do it to wean off of cigarettes. I know that cigarettes are not good for you, and all of the health concerns. I was like, okay. Maybe I’ll try this as an alternative to try and better my health”.

(18-year-old multi-race female)

#### 3.1.3. Stress Management/Relaxation

Participants often noted using ENDS to relax or manage stress:

“I just do it because when I get anxious or nervous… It helps me take a deep breath and let it out a little bit. Just helps me feel better”.

(18-year-old white transgender adult)

#### 3.1.4. Social Acceptability

Participants described that vaping is often more socially acceptable than smoking, largely because of the discreteness and less aversive smell:

“People don’t frown at you when you’re vaping. People do frown at you if you smoke a cigarette. When I vape there’s nothing said ‘cause you can’t smell it. You wouldn’t even know it unless you turned around and seen me vaping?”.

(36-year-old African American male)

#### 3.1.5. Cost

Participants described the long-term cost benefits of vaping compared to smoking:

“Well, a pack a day like six bucks. This [ENDS] is $40, but this last me longer than the pack of cigarette. The pack of cigarette probably last me like a day and then I have to buy another pack. This one last me three weeks”.

(49-year-old African American male)

Another noted,

“It’s a big purchase at first, but …the long-term cost is more affordable”.

(27-year-old female, other race)

#### 3.1.6. Flavors

Based on quantitative data (Table 1), the most commonly used ENDS flavor was fruit (76.7%), followed by menthol, mint, candy/dessert, and tobacco (20.0%–33.3%). Some participants described flavors as a key reason they enjoyed using ENDS (“I like to use my vape mainly because of the flavors. I don’t like the lingering smell of a cigarette. You smell disgusting. I like just to have the flavors” [21-year-old white female]). Others preferred ENDS that tasted more like their regular (menthol/tobacco) cigarettes:

“I prefer to just stick with the tobacco flavor. That way, I don’t have all that artificial stuff. Not to say the cigarettes is not artificial flavor, but we know that that liquid is artificially made”.

(55-year-old African American male)

### 3.2. Risk Perceptions

#### 3.2.1. Uncertainty

A prevailing theme was uncertainty due to a perceived lack of public health information regarding health risks of ENDS. Participants compared the lack of information about ENDS to the abundance of information on health consequences of smoking:

“I don’t know what to think. I don’t know what’s in that liquid just like I don’t know what’s in the nicotine, the chemical effect and everything. We hear about people having strokes from smoking cigarettes. I don’t know. You probably could have a heart attack from smoking electronic vapor cigarettes because we don’t know what the chemical is inside”.

(55-year-old African American male)

Another further expressed,

“The uncertainty is worse than knowing it’s negative. ‘Cause then I could at least make a decision based on the information. Now, I’m just waiting to find out”.

(18-year-old white male)

#### 3.2.2. Lower Perceived Relative Risk of ENDS

Many indicated vaping was less harmful than smoking (e.g., “the lesser of two evils”), though they were unsure of the degree:

“I know for a fact they are less harmful than cigarettes. I’m not saying that they’re healthy for you, by any means, but it is a lot better, I would say, than smoking cigarettes”.

(22-year-old Asian male)

#### 3.2.3. Concerns about ENDS Harms

Concerns about potential ENDS risks, including nicotine addiction, exploding devices, and cancer were common:

“I heard about one of ‘em blowing up…. I think about that sometimes…. That’s scary”.

(53-year-old African American male)

Participants commonly noted the presence of nicotine in ENDS as harmful:

“They say [ENDS] try to help you come down smoking cigarettes…. but that ain’t no better. It still have nicotine in it. It’s really harmful”.

(34-year-old African American female)

Similarly, ENDS were perceived as maintaining nicotine addiction:

“They might not have all the chemicals maybe, but it’s still nicotine. It’s still keeping you in this cycle”.

(19-year-old Asian female)

### 3.3. Satisfaction and Craving Reduction

Participants most commonly reported that ENDS managed nicotine cravings only to a certain extent and were not a fully satisfying substitute:

“About two months ago… I couldn’t afford to buy a pack of cigarettes and I took out my vaper and used it, but it wasn’t satisfying…. I can’t really explain it, but it doesn’t have that pull… like a real cigarette has”.

(58-year-old African American male)

Participants reiterated that ENDS were not a sufficient substitute for cigarettes although they were somewhat helpful for managing cravings when cigarettes were not allowed:

“When I don’t have a cigarette, it’s helpful. When I’m in public places, you can use that, but it really don’t satisfy you anyway, so you’re gonna go outside anyway, smoke that cigarette… It just didn’t seem like it was getting the craving of nicotine”.

(38-year-old white female)

Some noted that when they had intense cravings they turned to cigarettes (“If [the craving is] really bad I’ll choose a cigarette, but if it’s mediocre or isn’t that bad I vape” (36-year-old African American male). One man described:

“I’m vaping for more pleasurable, I guess. Cigarettes…I almost wanna say it’s a fact in my life. I have to have. Vaping, I don’t feel like—it’s not a have to have. It’s more of a want. Cigarettes, I need it. Vaping, to me, is fun. I wanna do it sometimes, but not a need”.

(41-year-old African American male)

On the other hand, others found ENDS helpful for managing cigarette cravings.

“I like the fact that [vaping] gets me near where cigarettes put me as far as alleviating the cravings and stuff. When I don’t have cigarettes, and if I had the liquid to go in it, that substitutes…. Before I even vaped, when I didn’t have cigarettes… I had to go outside, go to the store, get my cigarettes. Now, I don’t have to be in so much of a rush because I can just fire that electronic up and go on…”.

(55-year-old African American male)

### 3.4. Factors Affecting Choice to Smoke vs. Vape

Primary factors affecting whether participants smoked or used ENDS were availability of cigarettes, stress, and social situations. They also typically smoked in the presence of certain triggers (e.g., alcohol) or times of day (e.g., morning).

#### 3.4.1. Availability

Most used ENDS primarily when cigarettes were not available or allowed (“If I do not have a cigarette or a black and mild, I will use [ENDS] instead” (35-year-old African American female). Participants also noted finances as an important factor:

“Lately, with my financial situation, it’s been whatever I’ve had easiest access to… When my cartridge gets low, I tend to just stick to my regular cigarettes until I know I’m getting close to pay day”.

(41-year-old African American male)

Another described:

“In a financial situation where you don’t really have the money to buy cigarettes. You know you have this portable, rechargeable thing that you can use”.

(36-year-old African American female)

#### 3.4.2. Stress and Anger

Participants commonly preferred to smoke rather than vape in the context of stress and difficult emotions. One participant described, “[When I] get mad, got problems, I don’t want no e-cigarette. Give me a real cigarette” (34-year-old African American female). Another explained, “The cigarettes are for the stress. The vape is just for fun…. If I’m stressed I’ll smoke” (19-year-old multi-race male).

#### 3.4.3. Social Situations

Participants often used ENDS in situations with non-smokers, including children, but otherwise smoked cigarettes:

“Yeah, when my kids come home, my grandkids come over, I use the vape then. Other than that, I smoke cigarette”.

(African American male, age not reported)

### 3.5. Experiences with ENDS Use in Attempt to Quit or Reduce Smoking

Reducing or quitting smoking was a key reason why many participants used ENDS. Seventeen participants (56.7%) said they smoked fewer cigarettes since they started vaping:

“I use [ENDS] as a way to cut down and to eventually quit. The main reason because I see that it’s helped me cut down, so I tell myself that if it’s got me this far, I’m pretty sure that’ll it get me lower than this. I know that sooner or later, it’ll happen”.

(23-year-old African American male)

Conversely, many said their smoking habits had not changed since they started vaping and some attributed their inability to quit smoking to ENDS’ lack of craving reduction:

“First [tried ENDS] to see if I could quit smoking, but then when I realized it wasn’t enough nicotine in it, I went back to smoking. [ENDS helped] for a little bit for the first couple days. After that, I needed a cigarette”.

(38-year-old white female)

One participant felt that vaping could not help people quit if they had smoked for a long time:

“I’ve been smoking for 40 years… I would think that if you’ve been smoking less than a year and you did vaping, I think that would stop you. But if you’ve been smoking as long as I’ve been smoking, no”.

(58-year-old African American male)

Four participants reported smoking more cigarettes since they started vaping:

“The reason why I started vaporin’ was to try to get off the cigarettes, and it didn’t work out. It just made me want cigarettes a lot more…. before I started vapin’, I used to smoke a pack a day. I’m now at a pack and a half a day”.

(African American male, age not reported)

### 3.6. Factors to Increase Likelihood of Switching to Exclusive ENDS Use

Participants said they would be more likely to switch to exclusive ENDS use if the products were more affordable (“If I made more money, I would switch more to vaping”) or had more appealing flavors. They wished that ENDS had higher nicotine content, faster nicotine delivery and tasted/felt more drawn to smoking a regular cigarette (“If it tasted like a real cigarette, I would be down for it. It don’t taste like a real cigarette though” [38-year-old white female]). Another described that both lower cost and cigarette-like taste would be appealing:

“If they would lower the prices…If the name brand cigarette companies came out with a liquid that tastes just like the cigarette itself, that would be awesome”.

(36-year-old African American female)

Others emphasized the harmfulness of the products (“If they made the vaping products less harmful, then I would switch” [white male, age not provided]).

## 4. Discussion

Among this sample of low-income dual users of cigarettes and ENDS, the most prevalent reason for vaping was that it can be undertaken where smoking is not allowed. Participants emphasized using ENDS indoors, around non-smokers, and in other situations where smoking is not permitted, but otherwise they smoked cigarettes because it was more satisfying. They described vaping as more socially acceptable, discreet, and “easier to get away with” in public than smoking. Over half of participants reported reducing the number of cigarettes they smoked since they started vaping. However, current self-described use patterns were more consistent with complementing rather than substituting smoking with vaping. This dovetails with recent research showing that e-cigarette use, specifically when/where smoking is not allowed, is common among socially-disadvantaged dual users [28]. Factors that prevented participants from completely switching to ENDS included lack of satisfaction and uncertainty/concerns about ENDS health risks. Research is needed on whether specific ENDS devices and/or public health messaging is effective for helping low-income smokers switch to exclusive use of lower-harm products and eventually quit all tobacco products.

Other qualitative studies among e-cigarette users (not necessarily low-income) indicated that ENDS are perceived as less satisfying than cigarettes and that e-cigarettes are often used in situations where cigarettes are not available/allowed [29,30,31,32]. In interviews with e-cigarette users who used ENDS to quit smoking, those who relapsed to smoking (but not those who successfully quit) said e-cigarettes were not sufficiently satisfying [33]. In the present study, participants said they needed a “real cigarette” for high-stress situations, which is consistent with other qualitative studies of ENDS users [31,34]. Perceiving that ENDS do not sufficiently reduce cravings is a common reason for ENDS discontinuation [35]. ENDS device type and nicotine concentration may also play important roles [36,37,38]. In an experimental study of first-generation ENDS “cig-a-likes” versus tanks of varying nicotine concentrations, tanks with higher nicotine content were associated with greater satisfaction [39]. Some studies suggest that smokers who use refillable tanks or modifiable ENDS are more likely to transition to exclusive ENDS use [40,41], while others have found no association between device type and quitting smoking [42].

In other ENDS-related qualitative studies not focused on low-income smokers, participants also mentioned the importance of cost [34,43], but the theme was not as prominent as in the current study. Most participants in our study used tank systems, rechargeable, and/or refillable ENDS and noted that despite higher up-front costs they ultimately saved money compared to smoking. However, they also noted that lower ENDS costs might motivate them to switch to exclusive ENDS use. It will be important to determine whether certain ENDS device types are more conducive to quitting smoking, and if so, ensure that effective harm reduction tools are affordable and accessible for low-income smokers.

Uncertainty about the health effects of ENDS was common. Even participants who perceived ENDS to be less harmful than cigarettes expressed uncertainty (“I don’t have any proof”). Uncertainty about ENDS health effects has been common in other qualitative studies, although the general perception is that ENDS are less harmful than cigarettes [30,44]. In a study on comparative risk messages of ENDS versus cigarettes, participants viewed the lack of definitive information about ENDS health effects as negative and some viewed the uncertainty as indicating greater harm [45]. Another study found that uncertainty about the relative safety of ENDS compared to cigarettes is associated with lower likelihood of trying ENDS [46]. Uncertainty about ENDS health effects may be a major barrier to switching to exclusive ENDS use among smokers. Although ENDS are potentially less harmful than cigarettes, they are not harmless and there is still much to be learned given the lack of long-term studies [8]. Additionally, research is needed on the best ways to communicate absolute and relative health effects of ENDS versus cigarettes to convey the harm reduction potential of ENDS to current smokers who are otherwise unable to quit without enticing non-smokers to try ENDS.

Consistent with past research [34,47], we found that while some ENDS users deem flavors to be very important, others prefer non-flavored ENDS and/or had concerns about chemicals in e-liquid flavoring. More research is needed to understand potential health effects of e-liquid flavors and whether certain flavors help or deter quitting smoking. State and federal regulation of e-cigarette flavorings also remains a complex public health issue. Flavor bans may be crucial for preventing youth ENDS use, given that youth are particularly attracted to fruit and menthol/mint-flavored ENDS [48,49]. Conversely, flavors could be important for helping adults transition completely away from smoking [49]. Some research suggests that using fruit or candy/dessert-flavored ENDS is associated with higher likelihood of switching completely from smoking to ENDS compared to rejecting ENDS and continuing to smoke [50]. More studies are needed, particularly among low-income and other priority populations.

Research is also needed on other factors that might encourage low-income smokers to switch to lower-risk products and quit all tobacco products and to better inform tobacco cessation interventions for this population. Some studies [40,51,52], though not all [42], link daily (vs. non-daily) ENDS use to higher odds of smoking cessation. In the current study, less than half of participants used ENDS daily. If daily ENDS use does indeed facilitate smoking cessation and priority populations of smokers are less likely to use ENDS daily, tobacco disparities could be exacerbated [53]. Choi and Chen-Sankey [53] found that daily ENDS use was low, especially for non-Hispanic black smokers. Pending more research on associations between ENDS use patterns and quitting smoking, tobacco cessation counseling could include recommendations to use ENDS in ways that are more conducive to harm reduction.

There is a clear need for increased access to and awareness of evidence-based tobacco cessation services for low-income populations, who face numerous barriers to tobacco cessation [4]. Despite several no-cost, evidence-based programs such as the Tobacco Quitline, online/mHealth tobacco cessation programs, and in-person services, utilization is extremely low [14,54]. Moreover, tobacco cessation programs often fail to address issues of basic needs, including food and housing insecurity, money for necessities, and safety, which are critical barriers to cessation among low-income smokers [55]. Low-income populations also experience higher levels of acute and chronic stress, which hinders cessation [56]. Participants in the current study highlighted stress as a key reason for smoking rather than vaping. Policy, community, and individual-level interventions are needed to combat structural determinants of health that cause high stress and to promote adaptive stress management among low-income smokers. Smoking cessation interventions for low-income populations must also address use of multiple combustible tobacco products. Consistent with the current study, use of LCCs is disproportionately common among cigarette smokers living in poverty [57].

This study is limited by a small sample, and although not uncommon for qualitative research, generalizability may be limited. Data were collected in fall 2018, before widespread media attention about e-cigarette or vaping product use-associated lung injury (EVALI), which could alter smokers’ perceptions of ENDS [58]. By nature of our inclusion criteria (current dual users), people who already quit smoking by using ENDS were excluded. Although overall use patterns seem indicative of complementing versus substituting smoking with vaping, over half (57%) of participants reported reductions in smoking since starting to use ENDS and it is possible that they continued to reduce or quit smoking completely over time. However, the majority (73%) of participants were still smoking daily with an average of about 15 cigarettes per day. Research is needed on the health effects of varying degrees of reducing the amount smoked versus completely switching from cigarettes to ENDS. While reducing smoking frequency is associated with lower mortality risk, complete smoking cessation confers much greater benefit [59,60]. Moreover, there has been some concern that dual use might be associated with greater nicotine dependence, breathing difficulties, and cardiovascular disease risk compared to cigarette smoking alone [13,14,61]. Further study is needed to ascertain whether sustained dual users are delaying quitting completely and thereby increasing their duration of smoking, which research suggests may negate any potential benefit of reduced intensity of smoking [59,60,62,63]. While there is uncertainty about the long-term effects of using ENDS to reduce but not quit smoking, there are clear benefits to complete smoking cessation [14]. Large randomized controlled trials are needed to assess whether ENDS can help people from diverse backgrounds quit smoking.

## 5. Conclusions

This study adds to the dearth of literature on perceptions and use of ENDS among low-income cigarette smokers. Overall, this sample of dual users from low-income backgrounds reported complementing their smoking with ENDS use, particularly when smoking is not permitted or socially acceptable. Interviews provided an in-depth exploration of barriers to complete switching to exclusive ENDS use among low-income smokers, including lack of satisfaction for managing cravings and stress, uncertainty/concern about ENDS health effects, and cost. While some smokers were hopeful that they would eventually be able to stop smoking, others were disappointed that ENDS did not offer sufficient craving reduction or feel significantly drawn to smoking to be a satisfying substitute. Based on the current findings, low-income smokers might be more likely to switch to reduced-harm products that are affordable, perceived as substantially less harmful, taste and feel drawn to smoking a cigarette, and are more effective for reducing cravings. Continued research is needed to maximize any harm reduction potential of ENDS and ensure that these products do not contribute to worsening health disparities among low-income smokers.

## Figures and Tables

**Table 1:** Participant Characteristics (*N* = 30).

Demographic Characteristics	
Mean Age, years (SD, range)	30.2 (12.9, range 18–58)
Gender, *n* (%)	
Female	11 (36.7%)
Male	18 (60.0%)
Transgender	1 (3.3%)
Race/Ethnicity, *n* (%)	
Black/African American	14 (46.7%)
White	9 (30.0%)
Asian	2 (6.7%)
More than one race	3 (10.0%)
Other	2 (6.7%)
Hispanic/Latino	1 (3.3%)
Annual household income, *n* (%)	
USD 0–USD 2400	5 (16.7%)
USD 2400–USD 12,000	11 (36.7%)
USD 12,001–USD 18,000	3 (10.0%)
USD 18,001–USD 24,000	4 (13.3%)
USD 24,001–USD 36,000	2 (6.7%)
USD 36,001–USD 42,000	3 (10.0%)
USD 42,001–USD 54,000	2 (6.7%)
Education, *n* (%)	
Less than high school	4 (13.3%)
High school or GED	10 (33.3%)
Some college/technical school	10 (33.3%)
Associate’s degree	5 (16.7%)
Bachelor’s degree	1 (3.3%)
Cigarette Smoking Behaviors	
Cigarette smoking, *n* (%)	
Every day	22 (73.3%)
Some days	8 (26.7%)
Mean Cigarettes per day (daily smokers only) (SD)	14.9 (20.3)
Smoking Stage of Change, *n* (%)	
Intend to quit in the next 7 days	1 (3.3%)
Intend to quit in the next month	3 (10.0%)
Intend to quit in the next 6 months	6 (20.0%)
Intend to quit in the next year	7 (23.3%)
Intend to quit someday but not in the next year	11 (36.7%)
Never plan to quit	2 (6.7%)
Ends Use Behaviors	
ENDS Use, *n* (%)	
Every day	13 (43.3%)
Some days	12 (40.0%)
Rarely	5 (16.7%)
Mean Number of days used ENDS in past 30 days (SD)	19.7 (10.8)
ENDS product characteristics, *n* (%)	
Rechargeable	27 (90.0%)
Tank system	21 (70.0%)
Product can be refilled with e-liquid	24 (80.0%)
Cartridges or pods	16 (53.3%)
ENDS flavors used most often, *n* (%)	
Fruit	23 (76.7%)
Menthol	10 (33.3%)
Mint or wintergreen	9 (30.0%)
Candy or dessert	7 (23.3%)
Tobacco	6 (20.0%)
Coffee	4 (13.3%)
Alcohol or cocktail	2 (6.7%)
Spice	2 (6.7%
Use of ENDS in situations where smoking was not allowed, past 30 days, *n* (%)	27 (90.0%)
Other Tobacco Use Behaviors	
Current use of little cigars/cigarillos, *n* (%)	
Every day	10 (33.3%)
Some days	5 (16.7%)
Rarely	8 (26.7%)
Not at all	7 (23.3%)
Current use of traditional cigars, *n* (%)	
Every day	6 (20.0%)
Some days	2 (6.7%)
Rarely	6 (20.0%)
Not at all	16 (53.3%)
Hookah use, past 30 days, *n* (%)	10 (33.3%)
Smokeless tobacco use, past 30 days, *n* (%)	3 (10.0%)

Note: SD, standard deviation; USD, United States dollar; GED, general education development.

**Table 2:** Response Categories and Themes from Qualitative Interviews.

Response Category	Themes
Reasons for Vaping	Convenience (e.g., situations where smoking is not allowed) Efforts to reduce or quit cigarette smoking Stress management/relaxation Discreteness/social acceptability (e.g., lack of smell) Lower long-term cost compared to smoking Flavors
Risk perceptions	Uncertainty Lower perceived relative risk of ENDS Concerns about ENDS harms
Satisfaction and craving reduction	ENDS not a satisfying substitute; insufficient craving reduction Reduced craving for cigarettes
Factors affecting choice to smoke vs. vape	Availability (cigarettes not allowed, affordable, or otherwise available) Stress and anger Social situations Other triggers and contextual factors (e.g., alcohol, coffee, morning)
Experiences with ENDS use in attempt to quit or reduce smoking	Smoking less Smoking same or more
Factors affecting likelihood to switch to exclusive ENDS use/stop smoking	Cheaper/more affordable More appealing flavors Higher nicotine or faster nicotine delivery Taste/feel similar to smoking a regular cigarette Less harmful/safer

## Data Availability

The data presented in this study are available upon reasonable request from the corresponding author.
